# Determining the learning curve for a novel microsurgical procedure using histopathology

**DOI:** 10.1186/s12909-022-03407-6

**Published:** 2022-05-04

**Authors:** Amir Taher, Joanne Chow, Min Sung Kwon, Damien Hunter, Ania Lucewicz, Chameen Samarawickrama

**Affiliations:** 1 Royal Prince Alfred Hospital, 50 Missenden Rd, Camperdown, NSW 2050 Australia; 2 Centre for Vision Research, Westmead Institute for Medical Research, 176 Hawkesbury Rd, Westmead, NSW 2145 Australia; 3 School of Medical Sciences, University of Sydney, Camperdown, NSW 2006 Australia; 4 Westmead Clinical School, Discipline of Ophthalmology, University of Sydney, Darcy Rd, Westmead, NSW 2145 Australia; 5 University of Sydney, Darcy Rd, Westmead, NSW 2145 Australia; 6 Central Clinical School, Sydney University, Johns Hopkins Dr, Camperdown, NSW 2050 Australia; 7 Westmead Hospital, Cnr Hawkesbury Road and Darcy Road, Westmead, NSW 2145 Australia

**Keywords:** Corneal surgery, Wet laboratory training, Lamellar dissections

## Abstract

**Purpose:**

Wet laboratories are becoming an increasingly important training tool as part of a push to a proficiency-based training model. We created a microsurgical wet laboratory to investigate the utility of histopathology use in assessing surgical outcomes and determine the learning curve of a novel microsurgical procedure.

**Methods:**

A microsurgical wet laboratory was established using pig eyes to simulate the human cornea. Three novice surgeons and an experienced surgeon performed an anterior cornea lamellar dissection and the duration of the procedure was recorded. With the aid of histological analysis, the thickness and characteristics of the dissected graft was recorded. The number of attempts to complete the experiment, defined as three successful dissections with mean thickness below 100 μm, was documented.

**Results:**

The use of histopathology was highly successful allowing in-depth analysis of the dissected graft for each attempt. Trainees reached the endpoint of the study in 21, 26 and 36 attempts (mean: 28 attempts) whilst the corneal surgeon completed the experiment in 12 attempts (*p* = 0.07). Mean dissection thickness decreased over time for all participants. The mean dissection time for trainees was 10.6 ± 4.2 min compared to the corneal surgeon with a mean of 8.2 ± 3.1 min (*p* = 0.03).

**Conclusion:**

We propose a corneal wet laboratory model that allows for simple, efficient, and flexible microsurgical training. The use of histopathological analysis allows for careful graft analysis, providing objective feedback throughout the training exercise. Trainees demonstrated improvements in the three key aspects of the procedure: accuracy as evidenced by decreasing histological thickness, confidence by self-report and fluidity by decreasing duration of the procedure.

## Introduction

Technological advances and higher standards of patient care have led to the apprenticeship model for clinical training being questioned. Surgical training in particular has fundamentally shifted towards becoming a proficiency-based training model with high utilisation of simulated training [1, 2]. The need to balance trainee development whilst ensuring patient safety has led to a revolution in a range of simulation-based training tools including skills courses, virtual reality, and wet laboratory models across many surgical specialties [3].

The process of acquiring a new procedural skill in a proficiency-based training model requires a clearly defined endpoint or a set of skills to be identified prior to the commencement of training. The ideal simulation model allows for ongoing appraisal of trainee progress and is highly predictive of the trainee performing the procedure to an acceptable standard [4]. In hepatobiliary surgery for example, virtual reality training significantly reduced the rate of surgical errors in residents performing a laparoscopic cholecystectomy [5] whilst in orthopaedics, a dry shoulder model with pre-determined performance metrics was shown to be an effective training tool for shoulder surgery [6].

The development, validation and implementation of proficiency-based training models in Ophthalmology has been far slower compared to other surgical specialties [7]. A contributing factor is the microsurgical nature of the procedures which makes clearly defining endpoints much more difficult. In many surgical specialties, metrics and errors are relatively easily defined. For example, damaging surrounding structures and failure to adequately close tissue is a sentinel error in abdominal surgery, and is visible to the naked eye [5]. In Ophthalmology, and especially corneal surgery, assessing similar errors is far more difficult as the difference between a good and a poor outcome is a sub-millimetre difference that is not readily appreciable to the naked eye. Outcomes are detected post-operatively using imaging modalities that are not available in the intraoperative setting. Finally, there is no reliable metric available to use as a proxy for visual acuity, adding to the complexity of the situation.

In the past decade, corneal surgery has undergone a revolution with the introduction of lamellar keratoplasty techniques [8]. Lamellar approaches allow for selective replacement of diseased tissue while preserving healthy corneal tissue [9–13]. There are many advantages to justify the use of these new lamellar techniques, however they remain technically more demanding than traditional penetrating keratoplasty [13–17]. A major barrier to the uptake of these newer techniques is the steep learning curve associated with the increase in technical difficulty [18–20]. To date, this learning curve has not been studied in depth and methods to navigate this learning curve are needed. Part of the challenge stems from the unique nature and anatomy of ophthalmic microsurgery. The microscopic measurement of dissected specimens provides the opportunity to determine any defects or buttonholing which significantly affects the refractive potential.

This paper aims to address some of the challenges in microsurgical simulation based training by proposing a novel microsurgical wet-laboratory that uses histopathological analysis to assess outcome. We aim to determine the utility of our microsurgical wet laboratory set-up and investigate metrics to ascertain the learning curve of novice surgeons attempting a novel microsurgical procedure. The findings of this study will inform the feasibility of histopathology as a suitable tool to assess competency in wet-laboratory simulators.

## Methods and materials

### Participants

Three novice surgeons (comprising of three university students) with no microsurgical experience and one experienced corneal surgeon (with over 300 prior lamellar procedures) participated in the study.

### Wet laboratory setup

**Fig. 1:**
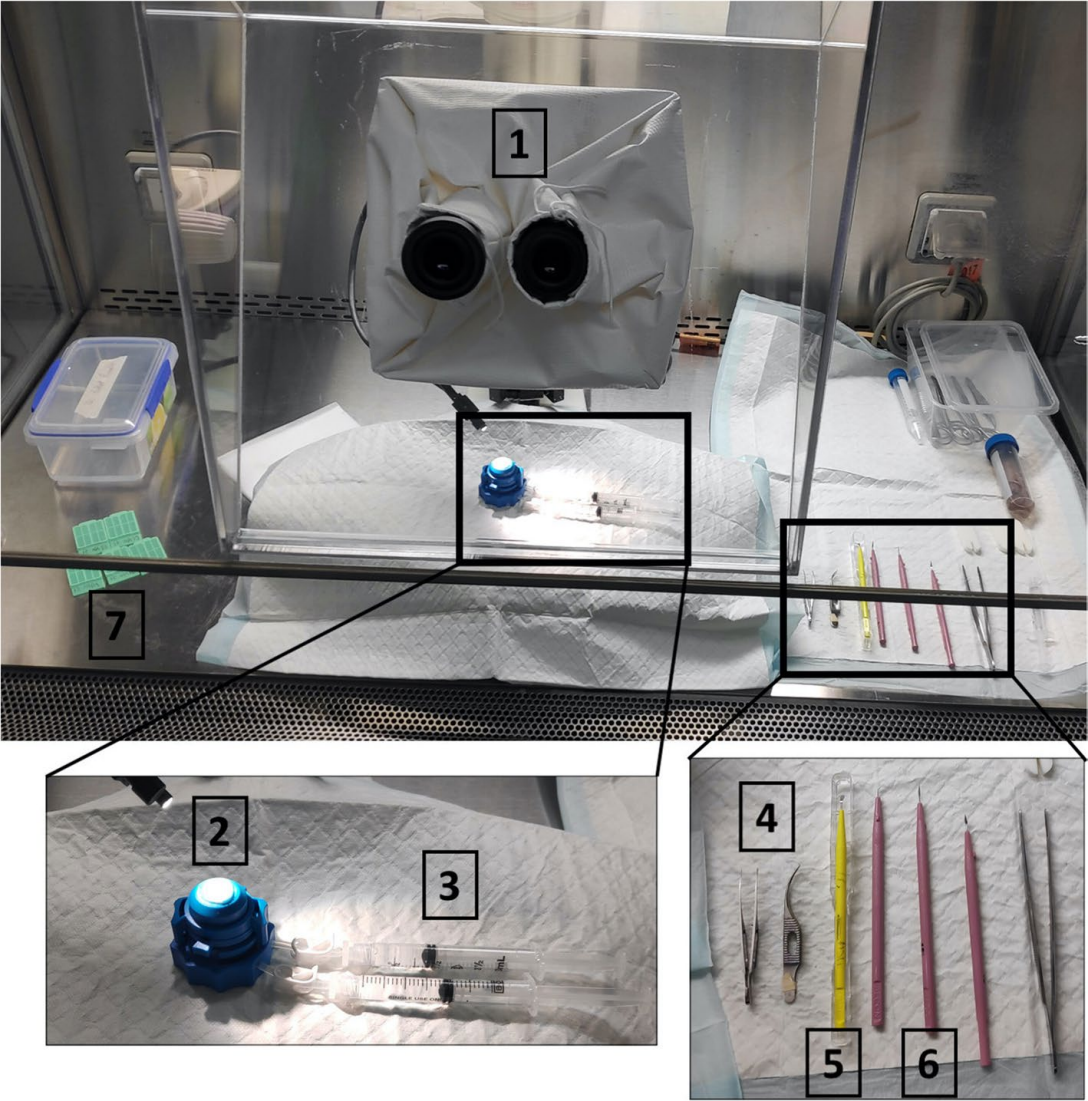
Microsurgical wet laboratory for corneal dissection. Equipment consisted of 1. Leica MZ6 Microscope, 2. Barron artificial anterior chamber, 3. Two 3-ml syringes, 4. Two Colibri micro-forceps, 5. Crescent Knife, 6. Fifteen degree Ophthalmic knife, 7. Slimsette Biopsy Cassette. All equipment was placed within a sterile Class II Biological Safety Cabinet

A microsurgical wet laboratory was established within a sterile Class II biological safety cabinet (Fig. 1), consisting of a microscope (Leica MZ6, Leica Microsystems, Wetzlar, Germany), artificial anterior chamber (Barron Precision Instruments, Michigan, USA) with two 3 ml syringes (Terumo, Tokyo, Japan), crescent knife (MANI, Utsunomiya, Japan), 15 degree ophthalmic knife (Alcon, Geneva, Switzerland) and two Colibri micro iris forceps (Barraquer, FCI, Paris, France).

Freshly harvested pig eyes were acquired 1 day post exenteration. Pig eyes were selected as their corneas are large, allowing for a surgical experience similar to that of human eyes. Corneoscleral buttons were prepared and stored in Dulbecco’s Phosphate Buffered Saline (Biowhittaker, Lonza, Switzerland) at 4 °C. The corneoscleral buttons were discarded if they were not used within 5 days, to ensure tissue integrity remained consistent across the study.

### Surgical technique

**Fig. 2:**
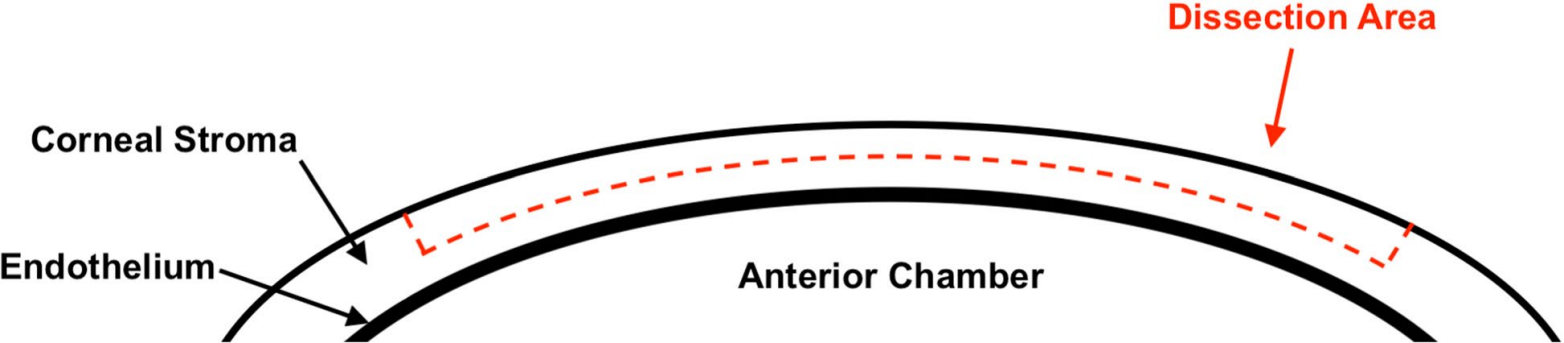
Simplified diagram of a lamellar corneal dissection

The aim of the surgical simulation exercise was to achieve a microsurgical dissection of the porcine cornea under 100 μm average thickness. The corneoscleral button was mounted on the artificial anterior chamber and the chamber was pressurised with two 3 mL syringes. The epithelium was removed with a surgical sponge. A partial thickness corneal incision was made close to the limbus with a 15° ophthalmic knife. A crescent blade was then used to continue the dissection across the entire cornea using a sharp dissection technique, avoiding buttonholes (Fig. 2). Each dissection was timed and once completed the tissue was immediately prepared for histology.

The procedure was explained to the novice surgeons in depth verbally, followed by the corneal surgeon performing the dissection as the trainees observed. The experiment was completed once three corneal dissections of an average depth below 100 μm was achieved by each participant. The trainees did not receive any intervening microsurgical experience.

This anterior corneal dissection technique was created for multiple reasons: it was easy for novice surgeons to conceptualise, especially after the demonstration; was straight forward to teach; and had the least number of variables in the dissection process to facilitate a smooth learning curve while maintaining the technical skills needed in microsurgery. Moreover, as the microsurgical wet-lab dissection was not identical to a standard corneal surgical procedure, the corneal surgeon also had to undergo a learning curve for the technique. The exercise intended to develop fundamental technical skills of corneal surgery, including the ability to remain within a corneal tissue plane, maintain a smooth tissue dissection plane mirroring the refractive potential of the tissue, ability to conduct dissections at less than 100 μm thickness, and manoeuvring and manipulating the hands as the surgeon works around the cornea.

### Histology and image analysis

Excised tissue was carefully flat mounted on a Slimsette Biopsy Cassette (Thermo Fisher Scientific, Massachusetts, USA) and fixed in 10% buffered formalin solution for 24 h, processed and paraffin embedded. Sections were hematoxylin and eosin stained, then slides were scanned at 20x magnification using a Nanozoomer Slide Scanner (Hamamatsu Photonics K.K., Japan).

The histological analysis was completed by a medical student who was trained in embedding and processing the tissue at the beginning of the study. The training time required to perform this analysis was less than 3 h in total. The process involved simple laboratory equipment including a generic Paraffin Embedding System and a Microtome.

**Fig. 3:**
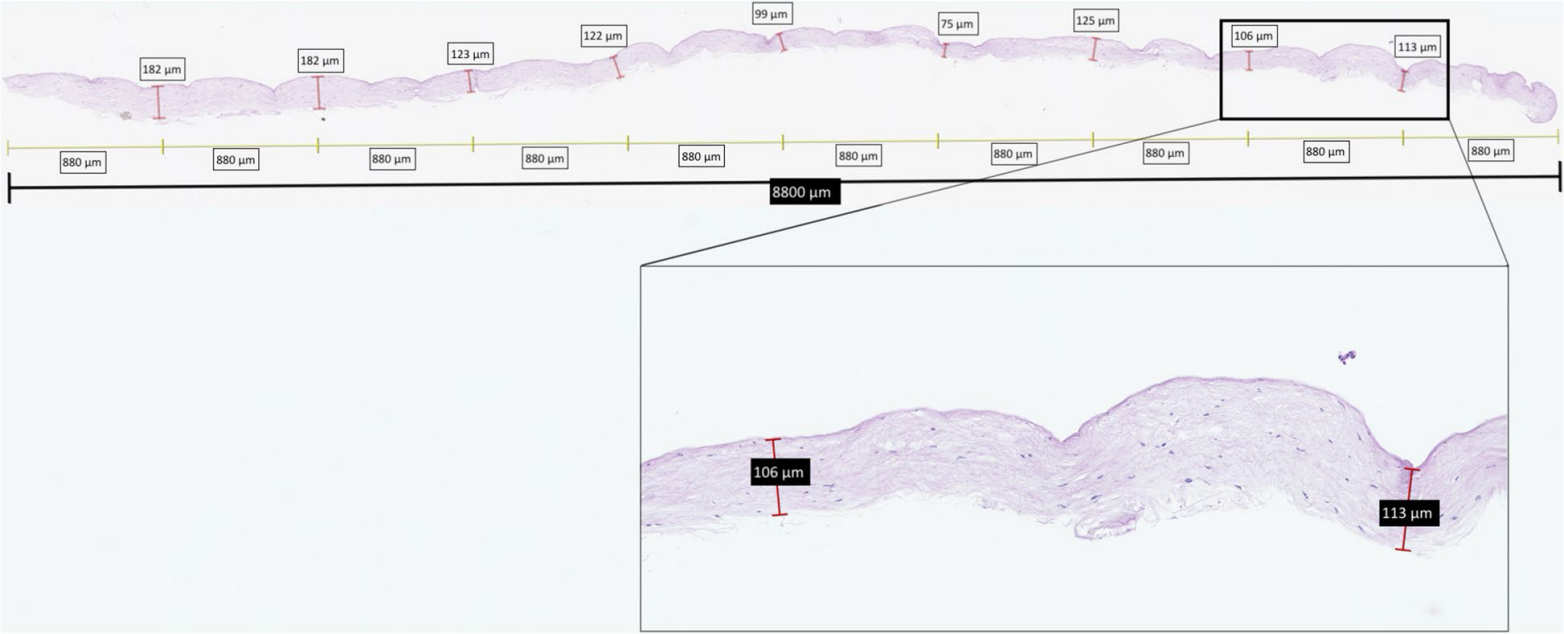
Sample dissection demonstrating the histology and measurements

Image analysis of scanned slides occurred using NDP.view2 software (Hamamatsu Photonics K.K., Japan). Tissue was divided into ten equal segments and the thickness was measured across nine evenly measured points along the length of the graft (Fig. 3). Average depth and standard deviation were calculated.

### Rationale

We chose to analyse our specimens histopathologically, as the real world difference in visual outcome is frequently determined by sub-millimeter thickness differences in dissections. The dissection thickness of under 100 μm was selected as it is the target thickness for Descemet Stripping Endothelial Keratoplasty (DSEK) transplants. Evidence highlights that sub-100 μm grafts have better visual outcomes than thicker transplants [21] and many surgeons and tissue banks use this thickness as their target benchmark.

Currently, there are no standardised simulations or training models available for corneal surgeons. For lamellar transplant techniques, surgical outcome is measured using vision 3 months post-transplant, and transplant thickness measured post-operatively using optical coherence tomography. Substandard dissections often lead to endothelial damage and an uneven transplant surface which ultimately leads to worse visual outcomes. The abnormal contour of the cornea leads to irregular astigmatism, which causes poor vision that glasses are inadequate at improving, and requires specialised contact lenses to optimise. The use of histopathology for accurate measurement of a graft in a surgical simulator setting is an excellent proxy as visual outcomes are largely dependent on graft thickness and variability.

### Feedback participant surveys

Regular feedback regarding dissection depth and quality was provided throughout the experiment as each dissected graft was processed and imaged. Surveys relating to the participant’s surgical confidence were completed at regular intervals during the experiment. The survey assessed each candidate’s confidence in achieving a dissection under 400, 300, 200 and 100 μm using a simple numerical scale of 1 to 10.

### Ethics

No ethics approval was needed as the experiment utilised ex vivo tissue purchased from a butcher and did not pertain to animals raised for research purposes. The students were able to participate without ethics approval as they were undertaking an approved research program within their university degrees.

### Statistical analysis

Data were analysed using SPSS software version 25 (SPSS Inc- Illinois, USA) and graphs were generated using GraphPad Prism version 8.0 (GraphPad Software, California, USA). Non-parametric Mann–Whitney U tests were used to compare results between trainees and the corneal surgeon. Pearson’s correlation was used to analyse the relationships between the frequency of attempts, and dissection thickness and time to completion of dissection. Line of best fit was selected for each data set based on the highest *r*^2^ value of linear or exponential models applied to data. Statistical significance was taken as *p* < 0.05.

## Results

### Dissection thickness and learning curve

**Fig. 4:**
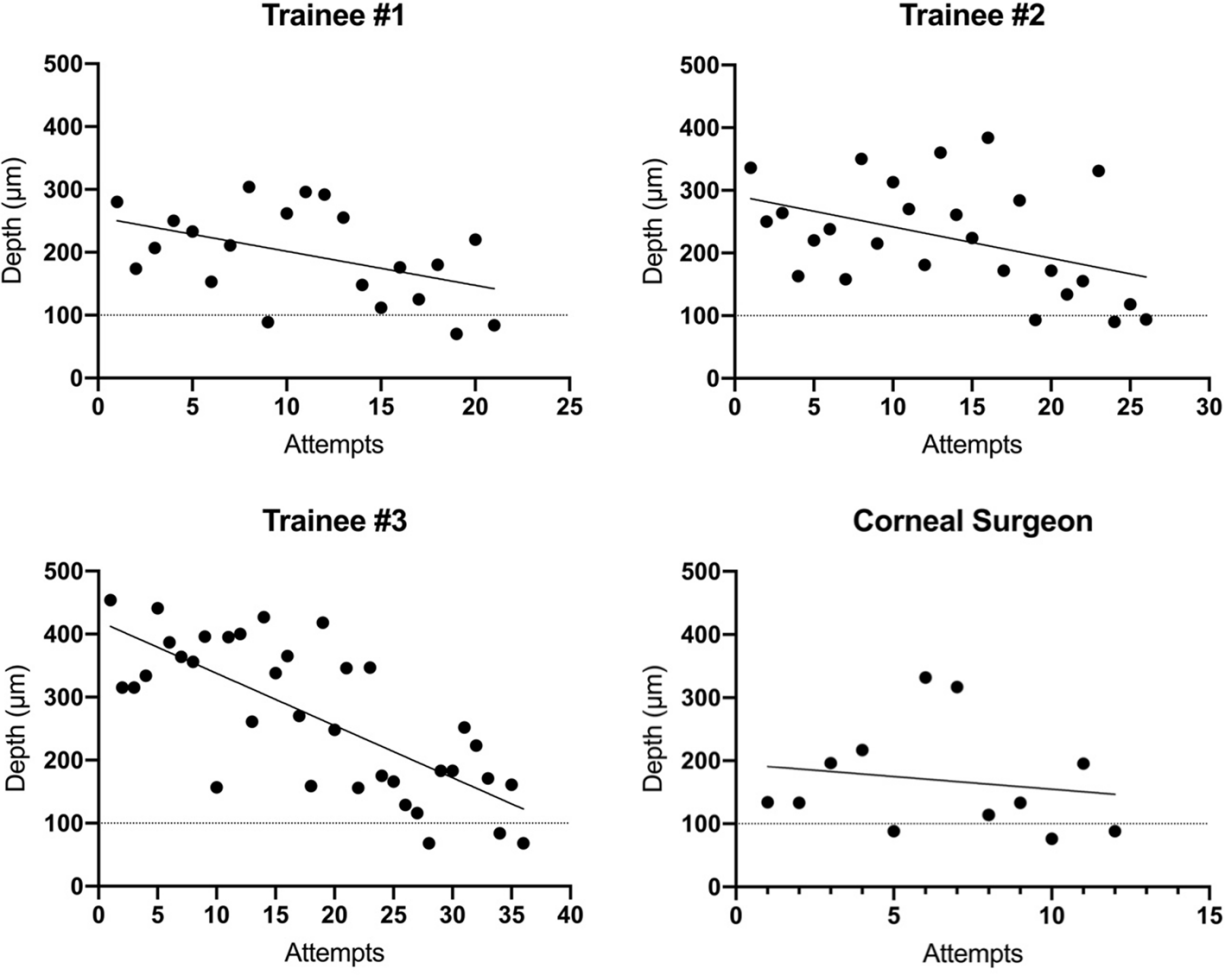
Graft thickness across all dissection attempts. A line of best fit shows an overall decrease in dissection thickness over time

All participants were able to perform three sub-100 μm dissections (Table 1). None of the grafts in our study had any significant defect (such as buttonholing) to report. Trainees reached study criterion in 21, 26 and 36 attempts (average 28 attempts), while the corneal surgeon reached the criterion in 12 attempts (*p* = 0.07). The learning curve for each participant is shown in Fig. 4. The graft depth for trainees ranged from 68 μm - 454 μm whilst the corneal surgeon’s range was 76 - 332 μm. Notably, there was no statistical significance (*p* = 0.67) between the graft thickness across the last 5 dissections for trainees (140 ± 43 μm) compared to the corneal surgeon (121 ± 47 μm).

**Table 1:** Summary of trainee performance compared to corneal surgeon

	Trainee #1	Trainee #2	Trainee #3	Trainee Average	Corneal Surgeon	***P*** Value
Attempts required to complete the experiment	21	26	36	28 ± 7.6	12	0.07
Mean duration of dissections (minutes ± standard deviation)	10.1 ± 3.0	11.3 ± 5.4	10.4 ± 4.1	10.6 ± 4.3	8.2 ± 3.1	**0.03**
Mean dissection thickness (μm ± standard deviation)	196 ± 74	224 ± 88	267 ± 117	236 ± 102	169 ± 86	**0.02**
Median dissection thickness (μm ± standard deviation)	207 ± 74	265 ± 88	222 ± 117	224 ± 102	133 ± 86	**0.02**
Mean thickness of the last five dissections (μm ± standard deviation)	136 ± 64	141 ± 64	158 ± 100	140 ± 73	121 ± 47	0.67

### Duration of dissection

**Fig. 5:**
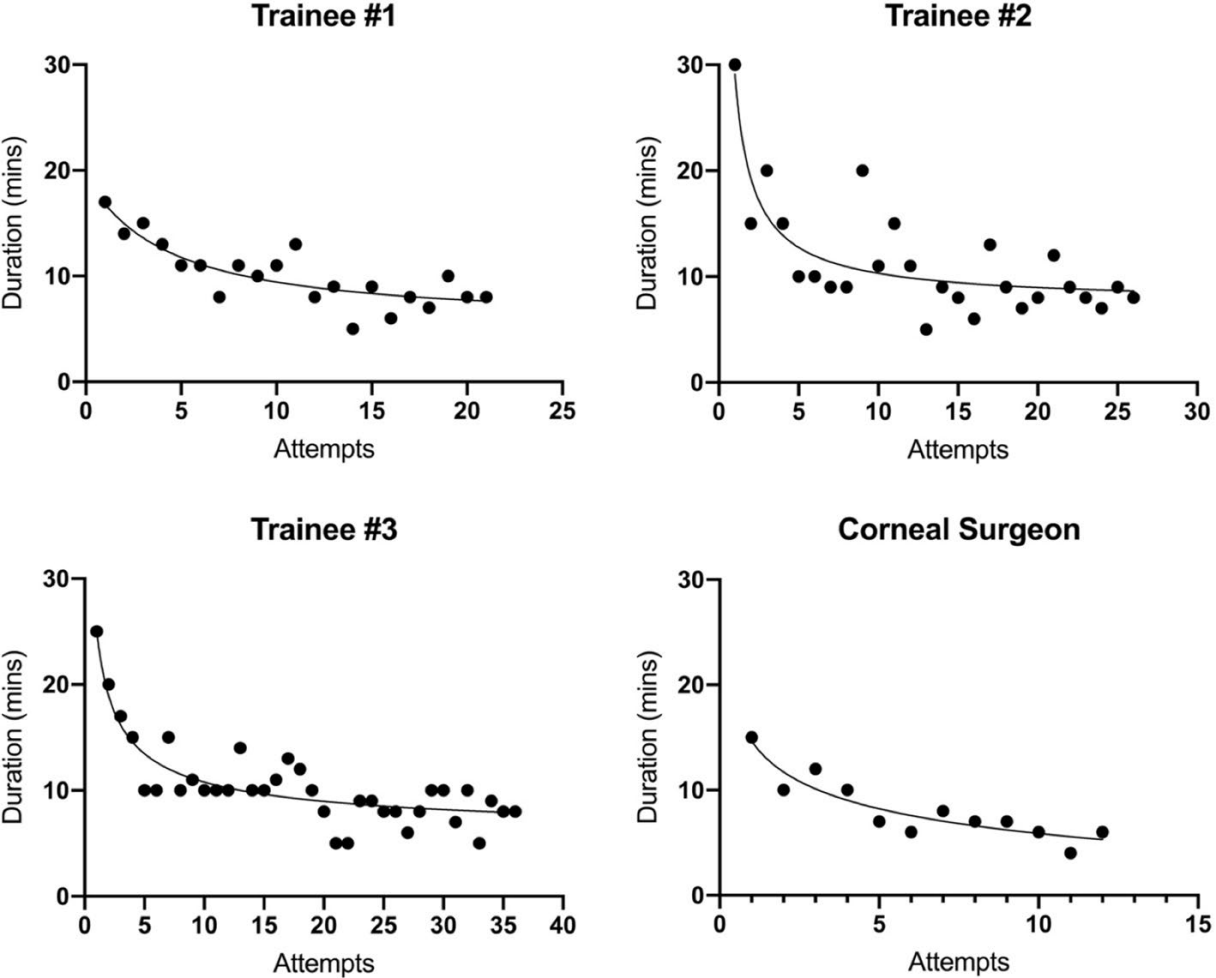
Surgical duration across all dissection attempts

The duration of dissections decreased over time for both trainees and corneal surgeon (Fig. 5). The median dissection time for trainees was 10 min (range 5-30 min) whilst the corneal surgeon had a median time of 7 min (range 4-15). The corneal surgeon was significantly faster at completing the dissections than trainees (mean dissection time 8.2 ± 3.1 min vs 10.6 ± 4.2 min; *p* = 0.03). The improvement in time was asymptotic, with the curve flattening as dissection durations approached 8 min for trainees and 6 min for the corneal surgeon.

For the final 5 dissections, there was no statistically significant difference in dissection duration between the corneal surgeon and trainees (*p* = 0.101).

### Confidence and evaluation

**Fig. 6:**
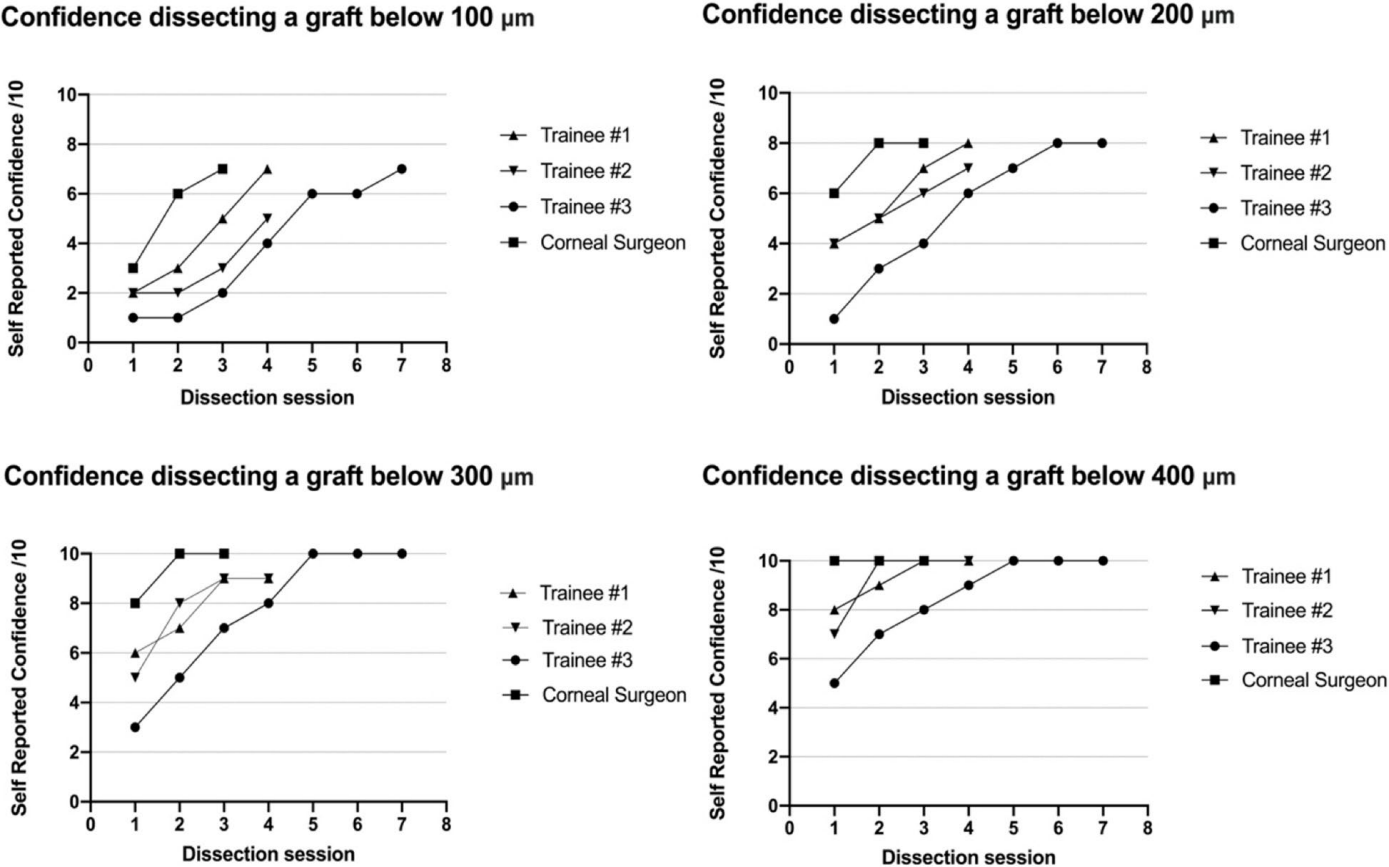
Self reported confidence prior to each dissection session across four different depth

All participants recorded an improvement in confidence with their dissection skills over time (Fig. 6). All participants noted an improvement in their perception of their surgical skills and their confidence attempting fine corneal dissections following their last dissection.

Trainees reported an overall increase of confidence in their microsurgical skills and believed that the training adequately prepared them to perform a similar lamellar corneal dissection. They valued the ability to undertake further dissections immediately following their histologically aided feedback. This allowed trainees to adjust their technique to improve their dissection quality and depth. No participant reported feelings of embarrassment or the need for additional training if the dissection depth or quality was not in line with their expectations or previous results. When asked about the use of wet laboratory training in other surgical procedures, all were of the opinion that simulation should become a routine part of medical education particularly in improving procedural skills.

## Discussion

Competency based education requires a shift from snapshot assessment of performance to frequent assessments over time to measure and ensure the attainment and maintenance of competency [22]. The generation of learning curves is an important step in the validation of competency based education and assessment [23]. With the aid of a validated learning curve, educators can more precisely target educational resources when designing curriculums and assess a candidate against the curve in order to identify individual learning needs and allocate educational resources accordingly.

Our proposed wet laboratory model successfully utilised histopathological analysis to assess the rate at which novice and experienced participants reached criterion and determined the learning curve of a novel microsurgical procedure in both these groups. The use of histopathology allowed for a unique insight into the evaluation of surgical technique, permitting the calculation of graft thickness down to the micron and review of the graft to check for surface defects or buttonholing.

Our described wet laboratory model of surgical education is in keeping with Ericsson’s concept of deliberate practice [24] as the teaching practice described is highly structured with explicit goals of improving performance in a specific task. It involves teaching by an expert followed by multiple levels of feedback on dissection depth and graft quality using histopathological analysis. The thickness threshold of 100 μm is closer to a mastery level threshold in clinical practice and defined as gold standard as the visual outcomes post-surgery are better in transplants below 100 μm thickness [21].

Mastery learning [25], refers to a form of competency based education where the learner is expected to achieve rigorous and fixed objective outcomes without any regard to the time needed to reach the mastery threshold. This approach often refers to a higher level of performance than competence alone. In our study, we defined it as 3 dissections under 100 μm in depth and demonstrated the learning curve for a new in a novice surgeon to be approximately 28 attempts compared to 12 attempts for a more experienced surgeon.

The most common learning curves tend to be S-shape indicating the change in learning rate as trainee becomes more experienced [23]. The curve gradually becomes asymptotic demonstrating maximal performance achievable in the task. In our wet laboratory, the duration of dissection and confidence performing the task had an asymptotic curve whilst the dissection thickness had a more linear shape, demonstrating effortful learning at a more constant rate (as determine by the slope) throughout the training process. This also suggests that, within ophthalmic surgery and especially anterior-segment surgery, learning outcomes should include a precise metric which can reflect visual refractive potential of the tissue – in this case being graft thickness measured by histopathology. We suggest that measurement of the learning curve by duration of dissection or participant confidence alone is limited, and reaching the asymptote before histopathological results indicated success. Therefore, metrics such as duration of procedure or participant confidence should be to be always supplemented with a metric that reflects graft refractive potential. We believe we have demonstrated histopathology as a feasible means of doing so.

One of the known barriers to the uptake of newer techniques in established surgeons is the steep learning curve associated with these complex advances [18–20]. This training model is suitable for experienced surgeons wishing to learn a new technique. We demonstrated that an experienced surgeon was able to learn a novel technique in < 15 dissections and though not directly translatable, anticipate a similar number of practice dissections may be needed to gain the basic skills for a procedure. Equally, established surgeons who have a reduced case load would benefit from this model in maintaining their microsurgical skills. Evidence from the Australian Corneal Graft Registry indicates that lower volume surgeons have poorer outcomes than higher volume surgeons in endothelial keratoplasties, indicating that a minimum number of cases are needed per year to maintain these refined surgical skills [26, 27]. Providing an alternative method of maintaining these hard learned skills is incredibly useful for surgeons, and can be used by regulatory bodies in assessing recency of practice.

The main limitation of this study is the small number of participants. However, most metrics measured achieved statistical significance, indicating conclusions can be drawn from this study. Further studies with larger sample sizes, as well as repeating the experiment 6 months later to determine if the skills gained were maintained, and if not how quickly could they be relearned, are planned.

In conclusion, we propose a wet laboratory model that allows for simple, efficient, and flexible microsurgical training. The use of histopathological analysis allows for careful graft analysis, providing objective feedback throughout the training exercise. This model is useful for trainees, fellows and corneal surgeons looking to improve or maintain their microsurgical skills and confidence. We demonstrated the learning curve for a new microsurgical procedure in a novice surgeon to be approximately 28 attempts compared to 12 attempts for a more experienced surgeon.

## Data Availability

All raw data and images for the study have been stored in a password protected computer and will be available upon request from corresponding author.
